# Anemia in myelofibrosis—prevalence, the *U2AF1* connection, new treatments

**DOI:** 10.1038/s41408-017-0032-9

**Published:** 2017-12-15

**Authors:** Ayalew Tefferi

**Affiliations:** Division of Hematology, Department of Internal Medicine, Mayo Clinic, 200 First St SW, Rochester, MN 55905 USA

Among 1000 consecutive patients with primary myelofibrosis (PMF) seen at the Mayo Clinic, 54% displayed a hemoglobin (Hgb) level of <10 g/dl and 38% were red cell transfusion dependent at the time of their referral^1^. The corresponding figures in patients seen at the time of their initial diagnosis were 38 and 24% and in those seen beyond 1 year of their diagnosis were 64 and 45%. In a subsequent study of 722 molecularly annotated patients with PMF^2^, anemia of any degree was documented in 628 (87%) cases at the time of their first referral to the Mayo Clinic, including 235 (33%) with severe (Hgb <8 g/dl), 98 (14%) with moderate (Hgb 8 to <10 g/dl), and 295 (41%) with mild anemia.

Pathogenesis of anemia associated with PMF is incompletely understood and contributing factors might include ineffective erythropoiesis that is intrinsic to the underlying clonal myelopoiesis, the functional consequences of marked splenomegaly, and the effect of inflammatory cytokines. We have recently discovered a strong and significant association between PMF-associated anemia and the presence of *U2AF1* mutation^2^; among 457 evaluable cases, the incidences of *U2AF1* mutations were 30% in patients with severe anemia, 18% moderate, 8% mild, and 3% in the absence of anemia; in multivariable analysis, anemia was associated with *U2AF1* mutations, absence of Janus-activated kinase 2 (*JAK2*)- or *CALR* type 1-like mutations, thrombocytopenia, older age, and constitutional symptoms. Experimental data suggest a connection between mutant *U2AF1* and altered hematopoiesis, accompanied by changes in pre-mRNA splicing. These observations suggest the potential value of drugs that target the spliceosome machinery for the treatment of PMF-associated anemia^3^.

Current prognostic models in PMF include anemia as a strong and independent predictor of shortened survival. These include the International Prognostic Scoring System (IPSS), dynamic IPSS (DIPSS), and DIPSS-plus. Among them, DIPSS-plus is the most comprehensive and employs eight risk variables: red cell transfusion need, Hgb <10 g/dl, platelet count <100 × 10^9^/l, leukocyte count >25 × 10^9^/l, circulating blasts >1%, age >65 years, constitutional symptoms, and unfavorable karyotype. Using these variables, four risk categories are currently considered: low (no risk factors), intermediate-1 (1 risk factor), intermediate-2 (2 or 3 risk factors), and high (4 or more risk factors). Application of DIPSS-plus in 967 consecutive patients from the Mayo Clinic resulted in median survivals of 1.8, 3.6, 7.8, and 17.5 years for high, intermediate-2, intermediate-1, and low-risk patients, respectively^1^. The prognostic value of anemia in PMF is underscored in the DIPSS-plus risk stratification system where a transfusion-dependent patient is automatically placed in the intermediate-2 or high-risk category, regardless of the presence or absence of the other risk factors. Transfusion need was also recently identified as an independent predictor of postsplenectomy survival in myelofibrosis^4^.

A number of drugs have shown activity in the treatment of PMF-associated anemia. These include erythropoiesis-stimulating agents, prednisone, androgen preparations, danazol, thalidomide, lenalidomide, pomalidomide, imetelstat, and momelotinib. Among these, the latter three are the most recent inductees, with only pomalidomide so far receiving Food and Drug Administration approval for the treatment of advanced multiple myeloma. In a recently published phase-3 study involving patients with myelofibrosis^5^, pomalidomide was not significantly better than placebo in providing relief from red cell transfusion dependency. These results were different from those of a previously reported non-controlled study^6^, where pomalidomide displayed antianemia activity, in a subset of *JAK2*-mutated patients with myelofibrosis, in the absence of marked splenomegaly or excess circulating blasts. Whether or not a more refined patient selection would have affected the outcome of the aforementioned pomalidomide phase-3 study remains to be determined. In the current edition of *Blood Cancer Journal*, investigators from China provide practically important information regarding the value of a three-drug combination of thalidomide, prednisone, and danazol in enhancing their individual activity for improving anemia in patients with myelofibrosis^7^.

Imetelstat is a 13-mer lipid-conjugated oligonucleotide that targets the RNA template of human telomerase reverse transcriptase. In a pilot study, the drug induced a complete or partial remission in 21% of patients with myelofibrosis, including molecular remission, reversal of bone marrow fibrosis, and abrogation of anemia^8^; imetelstat-induced responses were more likely to occur in the absence of *ASXL1* mutations and presence of *SF3B1* or *U2AF1* mutations. Momelotinib is a small-molecule, ATP-competitive inhibitor of JAK1 and JAK2. In a phase-1/2 study, momelotinib was able to accomplish not only what was expected from a JAK2 inhibitor therapy for myelofibrosis, which is palliation of symptoms and splenomegaly, but also improved anemia in a substantial fraction of patients^9^. The latter activity has since been linked to inhibition of activin receptor-like kinase-2-mediated hepcidin expression, thus promoting availability of stored iron and facilitating erythropoiesis^10^.
